# Arc-Induced Long Period Gratings from Standard to Polarization-Maintaining and Photonic Crystal Fibers

**DOI:** 10.3390/s18030918

**Published:** 2018-03-20

**Authors:** Flavio Esposito, Rajeev Ranjan, Stefania Campopiano, Agostino Iadicicco

**Affiliations:** 1 Department of Engineering, University of Naples “Parthenope”, Centro Direzionale Isola C4, 80143 Napoli, Italy; flavio.esposito@uniparthenope.it (F.E.); rajeev.ranjan@na.imm.cnr.it (R.R.); campopiano@uniparthenope.it (S.C.); 2 Institute for Microelectronics and Microsystems, National Research Council, 80131 Napoli, Italy

**Keywords:** long period gratings, optical fibers, optical fiber sensors, refractive index sensors, sensors fabrication, strain sensors, temperature sensors

## Abstract

In this work, we report about our recent results concerning the fabrication of Long Period Grating (LPG) sensors in several optical fibers, through the Electric Arc Discharge (EAD) technique. In particular, the following silica fibers with both different dopants and geometrical structures are considered: standard Ge-doped, photosensitive B/Ge codoped, P-doped, pure-silica core with F-doped cladding, Panda type Polarization-maintaining, and Hollow core Photonic crystal fiber. An adaptive platform was developed and the appropriate “recipe” was identified for each fiber, in terms of both arc discharge parameters and setup arrangement, for manufacturing LPGs with strong and narrow attenuation bands, low insertion losses, and short length. As the fabricated devices have appealing features from the application point of view, the sensitivity characteristics towards changes in different external perturbations (i.e., surrounding refractive index, temperature, and strain) are investigated and compared, highlighting the effects of different fiber composition and structure.

## 1. Introduction

The development of fiber gratings has produced a significant impact on research and development in telecommunications and fiber optic sensing. Fiber gratings are intrinsic devices that allow the control over the properties of light propagating within the fiber. They are used as spectral filters, as dispersion compensating components, and in wavelength division multiplexing systems [1,2]. The sensitivity of their properties to perturbations in the surrounding environmental conditions have conducted to extensive study of their use as fiber sensor elements, in addition, they have significant advantages, such as wavelength domain response, electromagnetic noise immunity, and compactness [3,4]. Fiber gratings consist of a periodic perturbation of the properties of the optical fiber, generally refractive index and/or geometry, and fall into two general classifications based upon the period of the perturbation. Short-period fiber gratings, or fiber Bragg gratings (FBGs), have a sub-micron period and couple the light from the forward-propagating core mode of the optical fiber to a backward, counter-propagating one [5,6]. The long-period gratings (LPGs), instead, have a period in the range 0.1–1 mm and promote coupling between the core mode and co-propagating cladding modes [7,8].

Concerning the LPG, the high attenuation of the cladding modes results in the transmission spectrum of the fiber containing a series of attenuation bands centered at discrete wavelengths, each one corresponding to the coupling with a different cladding mode. The exact form of the spectrum, and the resonance wavelengths of the attenuation bands, are sensitive to the period and length of the LPG, as well as to the local environment: temperature, strain, bend radius and the refractive index (RI) of the medium surrounding the fiber. The peculiar spectral features of LPGs have made them broadly used in many applications, ranging from telecommunications to sensing [9]. In particular, they represent one of the most important fiber grating technological platforms, to be employed in a number of chemical and biological applications because of their intrinsic sensitivity to surrounding RI (SRI) changes [10]. To further increase their use, several approaches have been proposed to achieve remarkable sensitivities, for example: cladding etching, coupling to higher order modes near their dispersion turning points, and multi-gratings configurations [11,12,13,14]. For LPGs coated with thin layers, having a refractive index higher than cladding, a huge sensitivity enhancement was found due to the so-called modal transition phenomenon [15,16,17,18]. As a result, applications of LPGs in several physical, chemical, and biological fields have been reported in a large number of works [17,18,19,20,21,22,23,24,25,26]. Moreover, their worldwide interest is confirmed by recent works concerning the fabrication in polymeric optical fibers (POFs). They hold particular importance for sensing applications, as they can withstand larger ranges of strain and have small Young’s modulus [27,28,29,30,31].

Different techniques are available for their fabrication, the most important being: UV irradiation [9], CO_2_ lasers [32], IR femtosecond lasers [33], mechanical deformations [34,35], and Electric Arc Discharge (EAD) [36,37,38]. The UV approach is one of the oldest but is still proposed for photosensitive fibers. While alternative and cheaper approaches, as CO_2_ lasers and EAD, were proposed afterwards due to their flexibility with important results. In fact, they can permit the inscription of LPGs in not photosensitive and unconventional fibers as well. LPGs obtained through a setup based on an electric arc were first presented by Dianov et al. [39]. From that work, the EAD technique has been widely investigated and review papers have been published by different researchers [36,37,40]. The perturbation is created by applying an electric arc to the fiber with a certain spatial periodicity [41,42]. The formation of the grating, in most cases, is attributed to fiber geometrical changes and to silica stress relaxation, moreover, the effects of dopant diffusion and micro-deformations were also investigated in specific cases [43,44,45,46,47,48]. In addition, some works discussed on the conditions leading to symmetry/asymmetry of cladding modes involved in the coupling mechanism [49,50]. Concerning the drawbacks of such technique, the most important limitation is the difficulty in the fabrication of LPGs with smaller periods, i.e., a limitation in the maximum cladding mode order. This is the reason why in recent years great efforts have been done in order to achieve gratings working in the dispersion turning point of phase-matching condition. Such goal was obtained recently, but in some cases, the results are still not satisfactory from the practical point view, i.e., long and weak gratings [51,52,53].

Mostly, EAD is a valuable approach when considering new fiber designs, with the aim of offering new perspectives in sensing applications [54,55,56,57,58]. On this line of argument, the fabrication of LPGs was achieved in optical fibers with different dopants, including: Al-doped and Al/Er-codoped [59], N-doped and S-doped [45], P-doped [60,61], pure-silica core with F-doped cladding [62,63,64,65], and radiation resistant [66,67] fibers. The fiber dopants play a vital role in changing the fiber physical and optical properties, such as the refractive index contrast, absorption/emission bands, melting properties, and so on. For example, an immediate implication is the possibility to strengthen or soften the fiber behavior towards radiations. Extensive studies have been reported in [66,67,68,69,70,71,72,73], concerning the radiation effects on commercially available silica-based optical fibers and unconventional ones doped with cerium, aluminum, erbium, fluorine, nitrogen, and phosphorus. In particular, the high radiation sensitivity of P-doped fibers, with respect to standard Ge-doped fibers, makes them suitable for radiation dosimetry [74,75,76]. On the contrary, pure-silica core fibers have been proven to show very low radiation sensitivity and thus the devices written in these fibers have been studied for applications in ionizing radiation environments [62,67,77]. These are just few of the examples remarking how the fabrication of sensors in fibers having different physical properties, represents a fundamental step for further success of fiber optic sensing technology.

In addition to the aforementioned class of optical fibers, there exist also a number with peculiar geometrical structures where the fabrication of LPGs was investigated as well. Among these, we recall for example polarization-maintaining [78,79], microstructured [80,81,82,83,84,85,86], and multi-core [87] fibers. In particular, polarization-maintaining fibers (PMFs) are designed such that birefringence is introduced, by means of asymmetric geometries and/or asymmetric refractive index profiles, in order to have two well-defined polarization modes propagating with very distinct phase velocities [88]. The most common PMFs are Bow-tie and Panda types, where the birefringence is created through stress. Such fibers can be employed for the realization of optical components as polarizers, couplers, and isolators [89]. In the last times, the fabrication of LPGs in such fibers has attracted attention exploring their use for the realization of polarization elements and mostly multi-parametric sensors [79,90,91,92,93]. Concerning microstructured optical fibers, particular attention has been focused on Hollow core Photonic crystal fibers (HC-PCFs), due to the unique characteristic of light propagation within the hollow core. This has a number of advantages such as lower Rayleigh scattering, reduced nonlinearity, novel dispersion characteristics, and potentially lower loss compared to conventional optical fibers [94,95]. In addition, HC-PCFs also enable enhanced light/material interaction, thus providing a valuable technological platform for ultra-sensitive and distributed biochemical sensing [96,97].

In this work, we report about our recent results concerning the fabrication of LPGs in several optical fibers, by means of EAD technique. In particular, the results are related to several silica optical fibers with both different dopants and geometrical structures, as: (i) standard Ge-doped SMF28; (ii) highly photosensitive B/Ge-codoped PS1250/1500; (iii) P-doped P-SM-5; (iv) pure-silica core DrakaSRH; (v) polarization-maintaining Panda PM-1300-XP; and (vi) hollow core HC-1550-02 fiber. An adaptive setup was arranged and the appropriate “recipe” was identified for each fiber presented, in terms of both arc discharge parameters and setup arrangement, for manufacturing LPGs with strong and narrow attenuation bands, low insertion losses and short length. As the fabricated devices have appealing features from the application point of view, the sensitivity characteristics towards changes in different external perturbations (i.e., surrounding refractive index, temperature, and strain) are investigated and compared, highlighting the effects of different fiber composition and structure.

## 2. Fabrication of Arc-Induced LPGs

Since LPGs are formed by periodic perturbation of the effective refractive index of the fundamental core mode, wavelength selective power coupling between fundamental core mode and higher order modes (or cladding modes in the case of single mode fiber) occurs. As matter of fact, the typical transmission spectrum of a LPG presents a number of discrete attenuation bands located at those wavelengths satisfying the phase-matching condition. In the simplest case, i.e., single-mode standard fiber, the resonant wavelengths are given by:

(1) $$\lambda_{i}=\left(n_{co}-n_{cl,i}\right)\cdot \Lambda$$

where $n_{co}$ and $n_{cl,i}$ are, respectively, the effective refractive index of core and *i*th cladding mode, while Λ is the period of the grating [9].

This section reports on the fabrication of LPGs in different optical fibers by EAD approach. In particular, the experimental setup and the physical mechanisms of gratings formation are discussed first. Subsequently, the details of the fabrication procedure, as well as the achieved results in different kind of fibers, will be presented and discussed.

### 2.1. Gratings Fabrication Based on EAD Phenomenon

**Figure 1:**
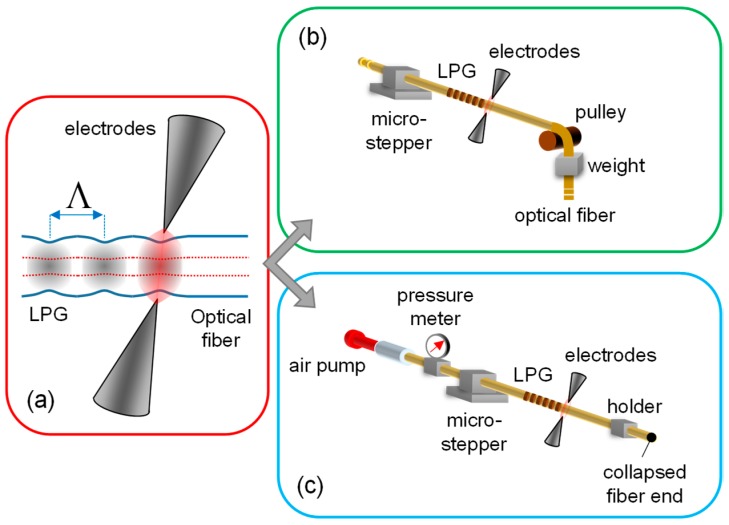
Schematic of Electric Arc Discharge (EAD) fabrication setup: (**a**) details of electrodes and tapered fiber; (**b**) configuration for solid silica fibers; (**c**) configuration for hollow core fibers.

As general point of view, the fabrication of gratings is achieved by inducing a periodic perturbation along the fiber, acting on physical and/or geometrical features of the original fiber. Basically, the EAD technique herein discussed is a step-by-step fabrication procedure allowing the creation of a sequence of single-perturbation or EAD-tapering, each one acting on both physical and geometrical features of the fiber. The heart of the fabrication procedure (responsible of the single-perturbation) is the arc discharge occurring between two electrodes, for heating the fiber in a controllable manner, as shown in Figure 1a. To this aim, we use the electrodes of a commercial fusion splicer (model Type-39 by Sumitomo Electric, Japan), which was modified to have the full control of the discharge parameters and the fiber alignment between the electrodes by working in open hood mode. The selection of the power and duration of the electrodes supply current, allows the control of the modulation strength and thus the LPG spectral features. Typical values are the arc power in range of 1–15 step (proprietary unit used by the manufacturer) and duration of 200–900 ms. In addition, to confine the effect of discharge along the fiber, the electrodes relative distance can be also changed from standard value of 2.0 mm to values in range 0.8–1.8 mm by a customization of the electrodes holders [38].

To obtain the single-perturbation in repeatable manner, the EAD procedure is usually combined with mechanical actions on the fiber under investigation. In particular, concerning the standard fibers or, in generic standpoint, concerning all silica fibers with solid core and cladding regions (leaving out all fibers with micro-structured regions), the procedure is applied to the fiber when it is subjected to constant axial tension. It can be easily achieved by means of a pulley and weight, as plotted in Figure 1b. The weight, and thus the applied force, represents a further freedom degree for the gratings fabrication; we typically used a weight of tens of grams. Finally, the fiber displacement of the grating period is operated by means of a micro-stepper, permitting to repeat the perturbation in the same conditions. The procedure is repeated until the desired spectral features of the LPG are obtained. It is worth highlighting that, since during the LPG fabrication both fiber ends are free, it is possible to have on-line spectral monitoring by combining the fabrication setup with the optoelectronic setup (see below) [63]. This fabrication platform was successfully adopted in several solid silica fibers, ranging from standard fibers [38,43,98], to specialty fiber with exotic dopants for specific application [59,60,62,63,86], and to polarization-maintaining fiber [78,79].

In some special optical fibers, such as the Hollow-core Photonic crystal fibers, the high fragility of the fiber does not allow the application of the same procedure for the formation of LPGs. In this case, the application of pulling tension during the discharge, through a weight, would produce a collapsing of the inner holes in fiber structure. Hence, a customized procedure based on a pressure assisted EAD technique was proposed for the first time in 2011 [81]. In particular, here the fiber was left free from axial stress on both sides of the arc region, via proper holders. Whereas a static pressure was applied inside the fiber holes (both core and cladding ones) during the EAD procedure as schematically plotted in Figure 1c, avoiding the collapsing of the fiber lattice structure. Similarly to the former case, after each EAD the fiber is displaced of the grating period by means of the micro-stepper, before applying the procedure again. It is import to remark that, in this case, it is not possible to have on-line monitoring of the spectral features during the grating fabrication.

Concerning the effects inducing the formation of the grating, it could be desirable to measure both geometrical changes and stress-induced RI changes, in order to identify the predominant one. However, while in principle the former can be easily measured, the study of the latter is very complex [37]. As a result, the conclusions of different works on the subject are not always in agreement. In particular, it was observed that the arc discharge relaxes intrinsic stresses in the fiber core and cladding but in regions that are larger than the grating period being, therefore, the refractive index modulation not enough to explain the grating formation [48]. On the other hand, an increase of the refractive index of the cladding and a decrease of the core-cladding difference were observed [47,99], making it possible. Unfortunately, the conclusions concerning the core region are not so straightforward and results may depend on the fiber and fabrication conditions as well [48]. Further studies revealed that the arc discharge is directional, possessing a temperature gradient that induces asymmetric micro-deformations in the fiber, which can account for the formation of the gratings [50]. Anyway it should be stressed that as the periodic modulation of the fiber was also pointed out as a potential origin of LPGs formation in [100], in other cases it was observed that it would require a severe deformation of the fiber cross-section (~17%) in order to obtain strong gratings [43].

**Figure 2:**
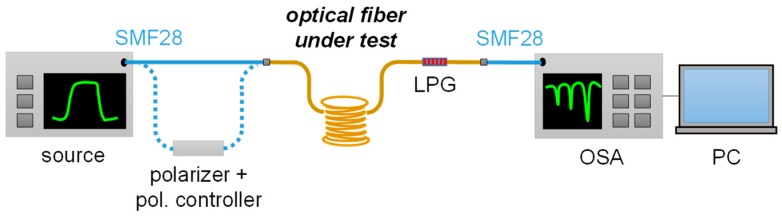
Optoelectronic setup for the measurement of Long Period Grating (LPG) spectrum.

Concerning the optoelectronic setup for the acquisition of LPG spectra, it is schematically reported in Figure 2. The illumination is provided by using a broadband SLED source (in wavelength range 1100–1700 nm), while the fiber transmitted spectrum with LPG is acquired by using the optical spectrum analyzer-OSA AQ6370B, Jokogawa, Japan (set to a resolution of 0.1 nm and sensitivity HIGH1), both connected to the fiber under investigation through SMF28 patchcords. Post-processing of data, based on centroid analysis [101], permits to identify the resonance wavelengths of the LPGs. Moreover, a polarizer (type ILP1550SM, Thorlabs, Newton, NJ, USA) and a polarization controller (FPC562, Thorlabs, USA) can be also connected after the source in the setup, in order to change the polarization of the light at the input of fiber under test [79], when requested.

### 2.2. LPG Fabrication in Solid-Silica Optical Fibers with Different Dopants

**Figure 3:**
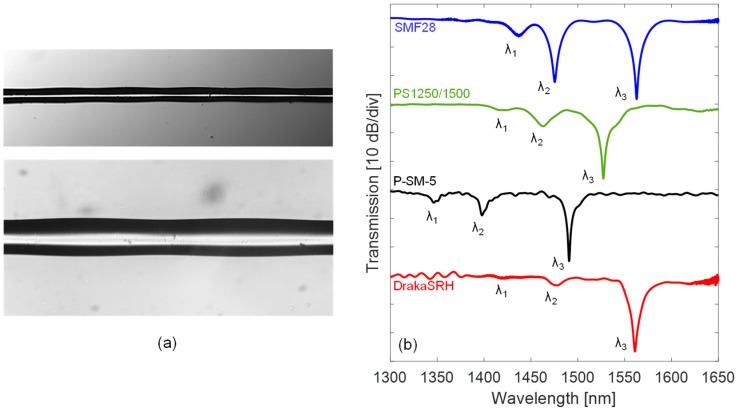
(**a**) Pictures of optical fiber with tapering effect due to arc-induced LPG; (**b**) Transmission spectra of LPGs in different single mode silica fibers.

The potentialities of the EAD fabrication technique have been first demonstrated by the realization of gratings in optical fibers having different dopant types in core and cladding regions. Most of the fibers considered here have low/null photosensitivity and thus it is not possible to realize LPGs by standard UV-technique. Here, the gratings have been achieved by using the setup reported in Figure 1b, where the fabrication parameters were selected depending on the hosting fiber (a 12 g weight was used in all the cases). In order to compare the results of different fibers, the following requirements were selected as target: (a) maximizing the power coupling for a relatively low order cladding mode (the 3rd order); (b) having the corresponding resonance wavelength around 1550 nm; (c) the final device with length below 25 mm; (d) insertion loss <0.7 dB. Following the details of fibers and LPGs, which are summarized in Table 1 as well:

(I) Standard Ge-doped core SMF28 fiber supplied by OZ Optics, having D_CO_ = 8.2 µm, D_CL_ = 125 µm, MFD = 10.4 ± 0.8 µm at 1.55 µm, and NA = 0.14. It is the standard single mode fiber for telecommunications. The grating was fabricated in this fiber with a period Λ = 628 μm, arc power = 1 step, arc duration = 430 ms, and length = 30Λ. The effect of arcs can be observed in the example pictures reported in Figure 3a, where the local tapering of the fiber is shown. While the resulting transmission spectrum of LPG in SMF28 is reported with blue line in Figure 3b, where three attenuation bands, $\lambda_{1}$, $\lambda_{2}$, and $\lambda_{3}$, associated to the first (LP_02_), second (LP_03_), and third order cladding mode (LP_04_), can be observed. In particular, concerning the $\lambda_{3}$ band, it is located at 1562.8 nm with very high depth of 28.3 dB.
(II) Highly photosensitive B/Ge-codoped core PS1250/1500 fiber by Fibercore, having D_CO_ = 6.9 µm, D_CL_ = 125 µm, MFD = 8.8 ± 10.6 µm at 1.55 µm, and NA = 0.12–0.14. The B/Ge combination gives extremely high photosensitivity whilst maintaining a relatively large mode field diameter, hence this kind of fiber is widely used for the UV inscription of fiber gratings. The LPG was fabricated with a period Λ = 640 μm, arc power = 15 step, arc duration = 750 ms, and length = 18Λ, resulting in $\lambda_{3}$ band centered at 1527.3 nm with depth of 26.1 dB, as from the green spectrum in Figure 3b.
(III) P-doped P-SM-5 fiber provided by Fiber Optics Research Center (FORC)-Russia, and having D_CO_ = 5.0 µm, D_CL_ = 126.1 µm, and NA = 0.18. It is specially designed for highly efficient Raman lasers and amplifiers operating in the 1.1–1.6 μm range, due to a higher value of the Raman shift as compared to standard one. The device was fabricated with a period of Λ = 700 μm, arc power = 1 step, arc duration = 420 ms, and length = 35Λ, resulting in $\lambda_{3}$ band positioned at 1490.7 nm with depth of 25.0 dB, and the spectrum is reported in Figure 3b with black line.
(IV) Pure silica core with F-doped cladding DrakaSRH fiber manufactured by Prysmian-Draka, having D_CO_ = 9.0 µm, D_CL_ = 125 µm, and MFD = 10.1 ± 0.5 µm at 1.55 µm. This fiber is optimized for use in highly radiative environments, due to pure silica core. The spectrum of LPG written in this fiber is reported in Figure 3b with red line, it was fabricated with a period Λ = 520 µm, arc power = 5 step, arc duration = 380 ms, and length = 30Λ. The $\lambda_{3}$ band is centered at 1561.9 nm with high depth of 26.6 dB.

**Figure 4:**
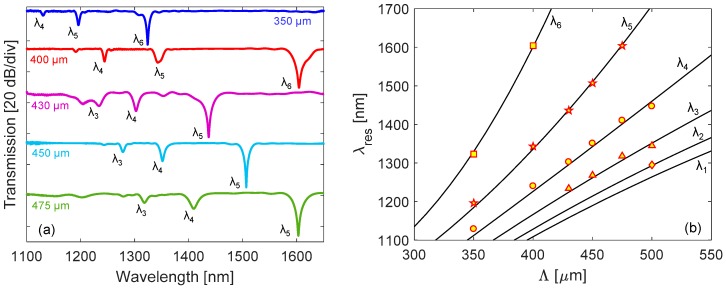
(**a**) Transmission spectra of LPGs in SMF28, with period Λ in range 350–500 μm; (**b**) Phase-matching curves, comparison between numerical (solid lines) and experimental values (diamond for $\lambda_{2},$ triangle for $\lambda_{3}$, circle for $\lambda_{4}$, pentagram for $\lambda_{5}$, square for $\lambda_{6}$).

As the fabrication of LPGs with lower period is an important aspect, due to their higher sensitivities, we have reported in Figure 4a the spectra of five gratings written in SMF28 with lower period Λ in range of 350–500 μm, where the excitation up to 6th order cladding mode can be obtained.

In order to support the fabrication phase and to validate the experimental results, a numerical model based on the coupled-mode theory (CMT) was developed [15]. This model allows determining the spectrum of a LPG, given the properties of the hosting fiber and grating, e.g., fiber geometry, core and cladding RI, external medium RI, grating modulation strength, period, and length [21]. In particular, we identified the gratings parameters related to our process [38]. As an outcome, we can calculate the resonance wavelengths of cladding modes versus grating period, as reported in Figure 4b. Here, they are also compared with the experimental values reported in Figure 4a, showing a good agreement.

### 2.3. LPG Fabrication in Polarization-Maintaining Panda Fiber

**Figure 5:**
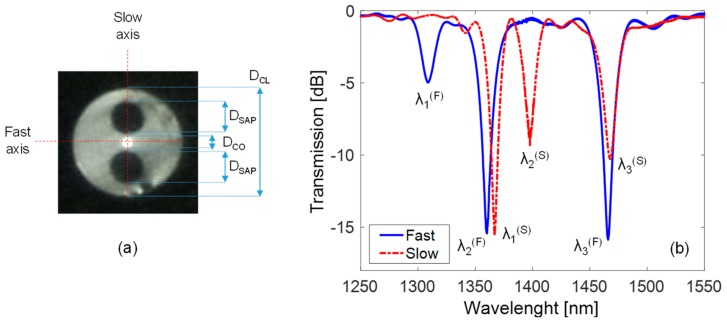
(**a**) Transverse section of Panda type polarization-maintaining fiber (PMF) model PM1300-XP; (**b**) Transmission spectra of arc-induced LPG in the fiber for fast and slow axis polarization.

Here we considered the polarization-maintaining PM1300-XP fiber from Thorlabs, in which the Panda type structure can be clearly observed from the picture reported in Figure 5a. The fiber has D_CO_ = 8.0 µm, D_CL_ = 125 µm, NA = 0.12, MFD = 9.3 ± 0.5 μm at 1.3 µm, and beat length < 4.0 mm at the same wavelength. As one can observe, along the slow axis the core region is comprised between two Panda-type stress-applying parts (SAPs) with diameter D_SAP_ = 36.0 μm and B-doping. The fast axis intercepts the fiber center and is perpendicular to the slow axis. The presence of the two SAPs produces significant material anisotropy in the fiber core due to the photo-elastic effects, with the result that the effective refractive indices of the core and cladding modes are different along fast axis and slow axis [88].

For the fabrication of LPGs in such fiber, the setup reported in Figure 1b is used, similarly to the other fibers investigated until now. Differently, for the acquisition of LPG spectrum, in the scheme of Figure 2 the polarizer and polarization controller are introduced after the source to manage the light polarization at the input of PMF. It is important to remark that here it is not required any control of the fiber orientation during fabrication, due to a good degree of symmetry of perturbation [78]. This is an important aspect, since in some cases, it was observed that the alignment between the perturbation and the fiber axes affects the fabrication process and thus the grating spectral features [102,103].

Hence, the LPG was fabricated in Panda PMF with period Λ = 600 μm, arc power = 1 step, arc duration = 360 ms, length = 42Λ, and 12 g weight (reported in Table 1). The spectra are reported in Figure 5b in the case of light linearly polarized along fast and slow axis, respectively. As one can observe, for each polarization the spectrum exhibits three attenuation bands due to the power coupling between core and different cladding modes [104]. Consequently, the phase-matching condition of Equation (1) has to be particularized for each polarization state, as:

(2) $$\lambda_{i}^{\left(P\right)}=\left(n_{co}^{\left(P\right)}-n_{cl,i}^{\left(P\right)}\right)\cdot \Lambda$$

where the apex (*P*) indicates the fast (*F*) and slow (*S*) polarization, and consequently $n_{co}^{\left(P\right)}$ and $n_{cl,i}^{\left(P\right)}$ are the effective indices of core mode and *i*th cladding mode for each polarization.

In particular, as one can observe from Figure 5b, in the case of fast axis polarization, the attenuation bands are located at $\lambda_{1}^{\left(F\right)}$ = 1308.8 nm, $\lambda_{2}^{\left(F\right)}$ = 1360.2 nm, and $\lambda_{3}^{\left(F\right)}$ = 1466.0 nm, with depth of 5.0 dB, 15.2 dB, and 15.8 dB, and corresponding to $n_{cl,1}^{\left(F\right)}$, $n_{cl,2}^{\left(F\right)}$, and $n_{cl,3}^{\left(F\right)}$, respectively. It can be noticed that such spectrum is similar to the one of LPG in SMF28 with similar period Λ reported in Figure 3b. As the light polarization is aligned with the slow axis, the attenuation bands are located at $\lambda_{1}^{\left(S\right)}$ = 1366.9 nm, $\lambda_{2}^{\left(S\right)}$ = 1397.5 nm, and $\lambda_{3}^{\left(S\right)}$ = 1467.6 nm, with depth of 15.3 dB, 8.6 dB, and 10.3 dB, and corresponding to $n_{cl,1}^{\left(S\right)}$, $n_{cl,2}^{\left(S\right)}$, and $n_{cl,3}^{\left(S\right)}$, respectively. The separation between resonant dips in the two polarizations is dependent on the birefringence of the fiber [90,92]. In our case, where the birefringence is high, the splitting can be also tens of nanometers, as one can observe from Figure 5b.

### 2.4. LPG Fabrication in Hollow Core Photonic Crystal Fiber

The standard EAD procedure was successfully applied to PCFs with solid core [86]. Differently, as the capillaries forming the holey structure of HC fibers are very thin, the use of standard procedure, where the discharge is combined with the application of axial tension (through a weight), leads to the localized collapse of the innermost ring of the cladding air holes, resulting in propagation losses [105]. To overcome these drawbacks, a combination of weak arc discharge and low static pressure inside the HC fiber holes can be used [81,82,83]. In particular, no axial tension is applied along the fiber, which is kept well aligned between the electrodes by proper holders and free from any mechanical tension. The arc discharge is provided when a static pressure is forced inside the fiber holes, resulting in a slight localized modification of the size and shape of core and cladding holes structure (without any collapsing, in this case) and thus a modulation of the effective refractive index.

**Figure 6:**
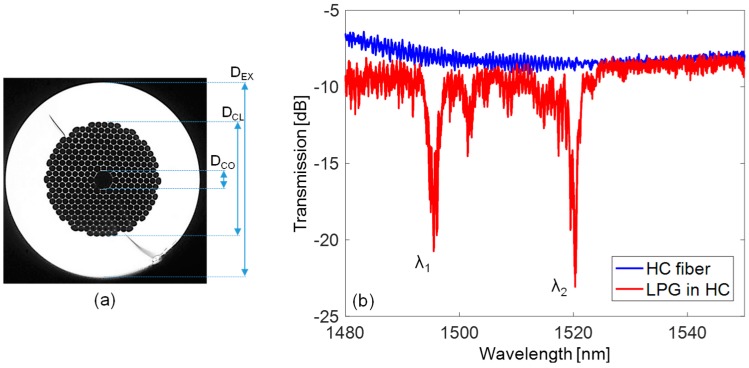
(**a**) Transverse section of HC-1550-02 Hollow core Photonic crystal fiber; (**b**) Comparison between transmission spectra of pristine HC fiber spliced with SMFs and with arc-induced LPG.

The Figure 6a illustrates the cross view of the hollow core fiber HC-1550-02, manufactured by NKT Photonics (Denmark). The fiber has external diameter D_EX_ = 120 µm and a microstructured cladding with D_CL_ = 70 µm. In particular, the cladding is formed by hexagonal holes arranged in a triangular lattice (with pitch of 3.8 ± 0.1 µm, from datasheet). Concerning the core, it is often represented by a circular-like hole with diameter D_CO_ = 11 ± 0.5 µm. Moreover, to fit the core shape the holes of the first cladding ring are slightly different from the rest of the cladding region. Finally, the fiber has MFD = 9 ± 1 µm at 1.55 µm and NA ~ 0.2.

For the fabrication of the LPG, the setup reported in Figure 1c is thus used, where the HC fiber is connected to a small air pump to force a static pressure inside the fiber holes, set to the constant value of 126 ± 0.1 kPa and monitored by a pressure meter. The arc discharges are provided also in this case by the electrodes of the same fusion splicer described above and the displacement is operated by the micro-stepper, while the absence of the weight, and thus any longitudinal force to the fiber, can be noticed. We highlight here that while one end of the fiber is connected to the air pump, the other end has core and cladding regions collapsed (by means of few arcs before the procedure) in order to stabilize the inner pressure during the procedure. As consequence, when the grating manufacturing is completed, the HC fiber with LPG is spliced to standard single mode fibers at both ends by manual procedure (optimized to reduce power losses) in order to measure the transmitted spectrum, as in Figure 2.

The LPG is fabricated with period Λ = 400 µm, arc power = 3 step, arc duration = 400 ms, and length = 25Λ (Table 1). The spectrum of such LPG is reported in Figure 6b, with respect to the one of pristine HC fiber spliced between SMFs, and it exhibits two attenuation bands. Here the power coupling occurs between the fundamental and higher order core modes [106]. In particular, as one can observe from Figure 6b, the attenuation bands are located at $\lambda_{1}$ = 1495.4 nm and $\lambda_{2}$ = 1520.3 nm, with depth of 9.4 dB and 11.9 dB, respectively. Finally, it is reasonable to believe that the background oscillations in the two spectra in Figure 6b are attributable to interference effects due to splicing with SMF and weakly excited higher order modes.

## 3. Sensing Features of Arc-Induced LPGs

In this section, we report about the sensitivity characteristics of the gratings presented above to surrounding refractive index, temperature, and strain. The sensitivity of a resonance wavelength $\lambda_{i}$ towards a parameter *ξ* can be obtained by applying the partial derivative with respect to *ξ* to the phase-matching equation. For example, considering the simplest Equation (1), it yields:

(3) $$\frac{1}{\lambda_{i}}\frac{\partial \lambda_{i}}{\partial \xi }=\frac{1}{\Delta n_{eff,i}}\frac{\partial \Delta n_{eff,i}}{\partial \xi }+\frac{1}{\Lambda }\frac{\partial \Lambda }{\partial \xi }$$

where $\Delta n_{eff,i}$ is the difference between effective refractive indices of core and cladding (respectively, higher order) modes for standard and PMF (for HC fiber). For the study of the LPG as sensor, the Equation (3) is then customized for the specific sensing parameter, as detailed in [9,10].

### 3.1. Characterization Setups

**Figure 7:**
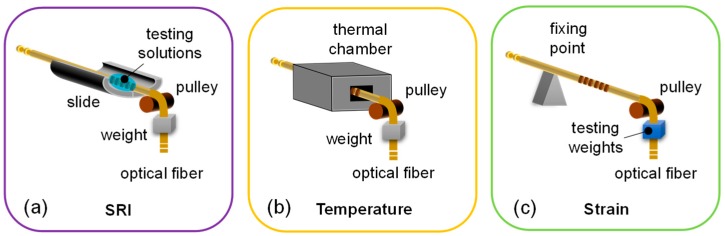
Schematic setup for the characterization of LPG towards: (**a**) surrounding refractive index; (**b**) temperature; and (**c**) strain.

The experimental setups for characterization to SRI, temperature and strain changes are reported in Figure 7. For SRI and temperature characterizations, the setups show a common structure aimed to keep the LPGs in constant strain state by means of a weight fixed to the fiber end (Figure 7a,b). Concerning the determination of SRI response, the scheme of Figure 7a permits to place the LPG into liquids with different refractive indices (i.e., aqueous solutions of glycerin at different concentration) ranging from 1.33 to 1.45, as measured at 589 nm by using a commercial Abbe refractometer with resolution of 10^−3^ RIU (Refractive Index Unit), keeping the temperature unchanged [38].

Similarly, as reported in Figure 7b, for temperature characterization the LPG is placed into a narrow channel (length is 10 cm and diameter is ~2 mm to ensure temperature uniformity) inside an aluminum block, which is housed on a controlled heater to change the temperature, for example as in our case from 25 °C to around 140 °C. As reference sensor, a commercial FBG-based sensor [3] is employed and positioned close to the LPG [60].

Finally, for the characterization towards strain, the setup is arranged as in Figure 7c, by applying in sequence several weights in range 0–85.0 g to the fiber hosting the LPG (temperature and SRI were kept unchanged during the process). To this aim, we used a pulley as the one used for the grating fabrication, and a FBG sensor as strain reference [79].

### 3.2. Sensitivity to Surrounding Refractive Index

**Figure 8:**
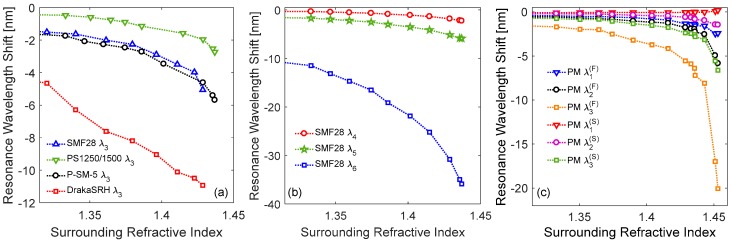
Resonance wavelength shifts towards surrounding refractive index (SRI) for LPGs in: (**a**) solid silica fibers with different dopants; (**b**) standard fiber with low period; (**c**) polarization-maintaining Panda fiber.

In this subsection, we report about the response to SRI changes of the LPGs presented in Section 2. First, we consider solid silica fibers with different dopants in Figure 3b, by focusing the analysis on the attenuation band $\lambda_{3}$ for all the gratings in order to better compare the results. The resonance wavelength shifts measured for the different fibers are thus reported in Figure 8a, with respect to their values in air. As one can observe, the increase in external refractive index produces a blue shift of the resonance wavelengths for all the gratings, with sensitivity increasing with SRI [38]. In particular, concerning the LPG in standard SMF28 fiber, a shift $\Delta \lambda_{3}$ of −1.51 nm was recorded for $n_{SRI}$ = 1.33 (i.e., water), which increases to −5.05 nm for $n_{SRI}$ = 1.43. Similar response was recorded by P-doped P-SM-5 LPG with values $\Delta \lambda_{3}$ of −1.75 nm and −4.62 nm for the same SRIs [60]. Differently, LPG exhibited lower sensitivity in B/Ge PS1250/1500 fiber with $\Delta \lambda_{3}$ of −0.47 nm in water and −1.96 nm at 1.43. Finally, the highest sensitivity was measured for the LPG in pure-silica core DrakaSRH fiber, with $\Delta \lambda_{3}$ = −4.65 nm for $n_{SRI}$ = 1.33 and $\Delta \lambda_{3}$ = −10.93 nm for $n_{SRI}$ = 1.43. In the latter case, the higher shifts are a consequence of the fluorine in cladding, since it lowers the cladding refractive index and thus the refractive index difference between this region and its surrounding, enhancing the sensitivity [63,107].

In order to highlight how the cladding mode order affects the sensitivity, we report in Figure 8b an example of SRI characterization of higher order attenuation bands ($\lambda_{4}$, $\lambda_{5}$, and $\lambda_{6}$) excited in a LPG fabricated in SMF28 with lower period Λ = 400 μm. As one can observe, when $n_{SRI}$ = 1.33, the measured wavelength shifts are $\Delta \lambda_{4}$ = −0.32 nm, $\Delta \lambda_{5}$ = −1.69 nm, and $\Delta \lambda_{6}$ = −11.47 nm, while they increase to $\Delta \lambda_{4}$ = −1.81 nm, $\Delta \lambda_{5}$ = −5.14 nm, and $\Delta \lambda_{6}$ = −30.81 nm when $n_{SRI}$ = 1.43. In particular, the high SRI sensitivity of sixth-order cladding mode makes it often used for chemical sensing [21,24]. Hence, we have observed that the sensitivity can be tuned by a proper selection of the fiber and fabrication parameters, depending on the application. Moreover, LPGs written in different fibers and thus having different properties can be combined for new sensing designs.

Figure 8c illustrates the wavelength shifts of the first three accessible cladding modes ($\lambda_{1}^{\left(P\right)}$, $\lambda_{2}^{\left(P\right)}$, and $\lambda_{3}^{\left(P\right)}$) of the LPG written in polarization-maintaining Panda fiber reported in Figure 5b, when considering fast and slow axis polarizations. Concerning the former case, the behavior is similar to the LPG in SMF28 [38,63]. In particular, considering $n_{SRI}$ = 1.33, wavelength shifts $\Delta \lambda_{1}^{\left(F\right)}$ = −0.43 nm, $\Delta \lambda_{2}^{\left(F\right)}$ = −0.58 nm, and $\Delta \lambda_{3}^{\left(F\right)}$ = −1.70 nm were measured. While for $n_{SRI}$ = 1.43, the wavelength shifts increased to $\Delta \lambda_{1}^{\left(F\right)}$ = −1.33 nm, $\Delta \lambda_{2}^{\left(F\right)}$ = −1.77 nm, and $\Delta \lambda_{3}^{\left(F\right)}$ = −5.54 nm. When the light polarization is aligned to the slow axis, a strong reduction in the sensitivity can be observed. In particular, for the same value of $n_{SRI}$ = 1.43, the shifts recorded for $\lambda_{2}^{\left(S\right)}$ and $\lambda_{3}^{\left(S\right)}$ are −0.6 nm and −2.39 nm, respectively. Moreover, the first attenuation band, $\lambda_{1}^{\left(S\right)}$, demonstrated practically insensitive to SRI. Such result can be related to the fact that along the slow axis, the cladding modes exhibit smaller evanescent waves in the external medium, $\partial n_{cl,i}^{\left(S\right)}/\partial n_{SRI}<\partial n_{cl,i}^{\left(F\right)}/\partial n_{SRI}$, which becomes practically zero for the first cladding mode, i.e., $\partial n_{cl,1}^{\left(S\right)}/\partial n_{SRI}\approx$ 0. This means that in these fibers, the presence of SAP regions results in the excitation of cladding modes with field strongly confined in fiber center (close to the core), and having small interaction with the surroundings. As matter of fact, this kind of LPG offers the possibility to tune the SRI sensitivity from zero to a maximum value by acting on the light polarization angle.

Finally, concerning the LPG in HC fiber, we would like to highlight that the resonant wavelengths showed negligible changes with SRI. In fact, according to [106], in such gratings the power coupling mechanism involves well-confined core modes, which can hardly interact with fiber external environment, and not cladding modes as for the other fibers presented here.

### 3.3. Sensitivity to Temperature

**Figure 9:**
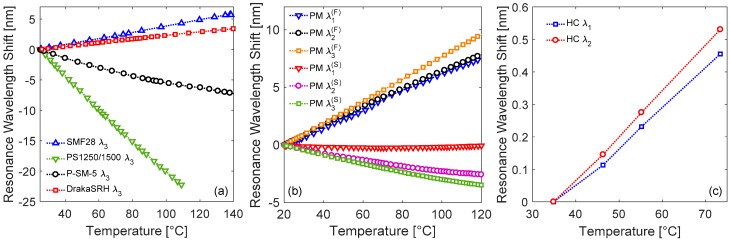
Resonance wavelength shifts towards temperature for LPGs in: (**a**) solid silica fibers with different dopants; (**b**) polarization-maintaining Panda fiber; (**c**) Hollow-core fiber.

Here we discuss the response of the same kind of LPGs to temperature changes, as acquired using the setup reported in Figure 7b. For this purpose, the results concerning the attenuation band $\lambda_{3}$ of the gratings in solid silica fibers are illustrated in Figure 9a. As one can observe, linear behavior are exhibited in all the cases, anyway the slope changes in magnitude and sign depending on the hosting fiber. The sensitivity values are summarized in Table 2, where the coefficient of determinations R^2^ are also reported, to evaluate the goodness of linear regressions. In particular, the sensitivity $S_{T,3}=\partial \lambda_{3}/\partial T=$ 50.6 pm/°C is recorded for SMF28 fiber, where the positive slope is associated to the term $\partial \Delta n_{eff,i}/\partial T$ in Equation (3) (while the contribution of the term ∂Λ/∂T can be neglected) and originates from the enhancement of the core thermo-optic coefficient α by the germanium [9]. In fact, typical values for this parameter are $\alpha_{{\mathrm{SiO}}_{2}}$ = 7.8 × 10^−6^/°C and $\alpha_{\mathrm{Ge}-{\mathrm{SiO}}_{2}}$ = 7.97 × 10^−6^/°C for pure-silica and Ge-doped silica, respectively [10]. While the grating in DrakaSRH exhibits lower sensitivity $S_{T,3}$ = 29.8 pm/°C, since the fluorine depresses the thermal coefficient of silica, anyway the overall response is still positive as the fluorine is in the cladding region [62,63]. Differently, the presence of phosphorus and boron produces a negative response with temperature increasing, with sensitivities $S_{T,3}$ of −58.0 pm/°C and −265.1 pm/°C recorded for P-SM-5 and PS1250/1500 fiber, respectively. According to Equation (3), to have a negative temperature behavior, the term $\partial \Delta n_{eff,i}/\partial T$ must be negative, i.e., a reduction in the thermal sensitivity of the effective refractive index of the core mode as compared with the one related to cladding mode is necessary. Indeed, due to the large negative thermo-optic coefficient of phosphorus [108], a reduction in the core thermo-optic coefficient is expected, which can be estimated to be equal to $\alpha_{P-{\mathrm{SiO}}_{2}}$ = 7.3 × 10^−6^/°C [60]. Similarly, also the boron codoping produces a significant reduction in the core thermal coefficient, which can be in range $\alpha_{B/\mathrm{Ge}-{\mathrm{SiO}}_{2}}$ = 7.3–7.7 × 10^−6^/°C [10]. As an overall assessment, it was observed that the kind of dopant plays a fundamental role concerning the temperature response, affecting both its magnitude and sign.

We now focus the attention on the resonance wavelengths of cladding modes excited in Panda fiber, considering as usual the two main orthogonal polarizations. As one can observe, in fast polarization the attenuation bands experience red shifts with temperature increasing, similarly to LPGs in standard fiber. In particular, the responses exhibit a linear behavior with sensitivities $S_{T,1}^{\left(F\right)}$ = 76.8 pm/°C, $S_{T,2}^{\left(F\right)}$ = 77.7 pm/°C, and $S_{T,3}^{\left(F\right)}$ = 96.3 pm/°C (associated to $\lambda_{1}^{\left(F\right)}$, $\lambda_{2}^{\left(F\right)}$, and $\lambda_{3}^{\left(F\right)}$, respectively), which are higher than in standard fiber. When the polarization is changed to slow, the responses change completely. In this case, shifts towards shorter wavelengths are measured with temperature increasing. In particular, concerning the $\lambda_{2}^{\left(S\right)}$ and $\lambda_{3}^{\left(S\right)}$, the sensitivities are $S_{T,2}^{\left(S\right)}$ = −25.6 pm/°C and $S_{T,3}^{\left(S\right)}$ = −34.9 pm/°C, respectively. Differently, the $\lambda_{1}^{\left(S\right)}$ is practically insensitive to temperature changes. It is reasonable to believe that the negative temperature response is due to SAP regions and, thus, affecting only the spectrum in slow polarization. For example, in comparison with other LPG in Panda fiber from [92], we measured a slightly lower sensitivity with respect to the value of 129.1 pm/°C measured for fast axis case, probably due to different cladding mode order. While the resonance wavelengths in slow axis state show slightly higher sensitivities than the value of −36.6 pm/°C reported in the same work.

Finally, Figure 9c illustrates the temperature induced wavelength shifts for the modes $\lambda_{1}$ and $\lambda_{2}$ of the LPG arc-induced in HC fiber. As one can observe, both the attenuation bands exhibit red shifts with temperature increasing. In particular, the modes have sensitivities $S_{T,1}$ = 11.9 pm/°C and $S_{T,2}$ = 13.8 pm/°C, respectively. Such values are lower than those of Figure 9a, since in HC fibers the temperature dependence is associated mostly to the term ∂Λ/∂T in Equation (3), since the term $\partial \Delta n_{eff,i}/\partial T\;\approx \;0$, because the fiber is made by single material and air core. Whereas, if compared to other LPG in HC fiber from [106], the sensitivity is higher, probably caused by the different fabrication techniques and order of the attenuation bands.

### 3.4. Sensitivity to Strain

In this subsection, we report the results concerning the characterization with respect to the last parameter under investigation, i.e., strain, which were performed using the setup in Figure 7c. In this case, the sensitivities are summarized in Table 3.

**Figure 10:**
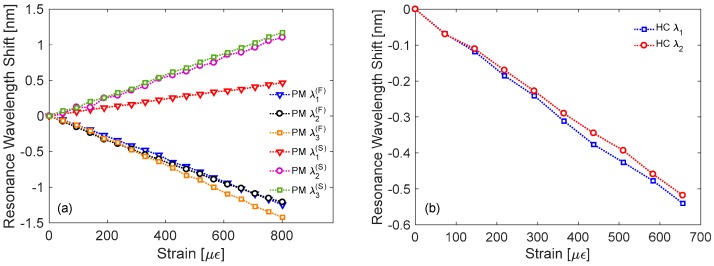
Resonance wavelength shifts towards strain for LPGs in: (**a**) polarization-maintaining Panda fiber; (**b**) Hollow-core fiber.

Concerning the LPG in Panda PMF, as evident from Figure 10a the wavelength shifts of resonant dips for fast axis and slow axis polarization exhibit opposite signs, as occurred for temperature response. Resonant wavelengths related to the fast axis polarization move to lower wavelengths with strain increasing, similarly to standard fiber [9]. Relatively higher sensitivities $S_{\varepsilon ,1}^{\left(F\right)}=\partial \lambda_{1}^{\left(F\right)}/\partial \varepsilon$ = −1.57 pm/μɛ, $S_{\varepsilon ,2}^{\left(F\right)}$ = −1.51 pm/μɛ, and $S_{\varepsilon ,3}^{\left(F\right)}$ = −1.82 pm/μɛ were measured, if compared with the value of −1.36 pm/μɛ from other LPG in Panda fiber [92]. Instead, the resonant wavelengths related to the slow axis polarization exhibit red shifts with slightly lower sensitivities as compared with fast axis case. In particular, such sensitivities were $S_{\varepsilon ,1}^{\left(S\right)}$ = 0.57 pm/μɛ, $S_{\varepsilon ,2}^{\left(S\right)}$ = 1.41 pm/μɛ, and $S_{\varepsilon ,3}^{\left(S\right)}$ = 1.47 pm/μɛ.

The results concerning the strain sensitivity of the attenuation bands of HC-LPG are reported in Figure 10b. They exhibit linear shifts towards lower wavelengths with $S_{\varepsilon ,1}$ = −0.82 pm/μɛ and $S_{\varepsilon ,2}$ = −0.78 pm/μɛ. These sensitivity values are very close to the sensitivity of −0.83 pm/μɛ of the HC-LPG realized in [106] in the same fiber. Differently, they are lower than the value of −2.76 pm/μɛ reported in [86] for a LPG arc-induced in solid core PCF, probably due to the different fiber geometry and thus strain response.

## 4. Conclusions

In this work, we have reported about our recent results concerning the fabrication of LPGs in several optical fibers by means of the EAD technique. We have considered the following fibers models, presenting both different dopants and geometrical characteristics: standard SMF28, B/Ge-codoped PS1250/1500, P-doped P-SM-5, pure-silica core radiation hardened DrakaSRH, Panda PM-1300-XP, and PCF HC-1550-02. An adaptive setup was presented, which can be used for the fabrication and characterization of the gratings in all these fibers. For each fiber, we identified the proper fabrication parameters in order to write LPGs with strong and narrow attenuation bands, low insertion losses and short length. Moreover, the response of these LPGs towards changes in SRI, temperature, and strain were investigated and critically presented. The choice of dopant type (including, but not limited to, Ge, B, P, F) plays an important role also in tuning the sensitivity of LPGs to different parameters. Moreover, the combination of different gratings permits new sensing designs. For example, concerning the HC-LPG here presented, as the response of such grating to curvature is very low [82], the results indicate that it may be used as a strain sensor without cross-sensitivities to curvature and external refractive index, and reduced dependence upon the temperature. Whereas, the unique properties of LPG in Panda fiber can be used for the realization of a 3-parameter sensor for the simultaneous measurement of SRI, temperature, and strain, based on a single fiber hosting a single device.

## Figures and Tables

**Table 1:** LPGs fabrication parameters and fiber types.

Fiber Model	Fiber Type	LPG Λ [μm]	Arc Power [Step]	Arc Time [ms]	Weight [g]	Number of Arcs
SMF28	Ge-doped core	628	1	430	12	30
PS1250/1500	B/Ge-codoped core	640	15	750	12	18
P-SM-5	P-doped	700	1	420	12	35
DrakaSRH	Pure-silica core, F-doped cladding	520	5	380	12	30
PM1300-XP	PM Panda	600	1	360	12	42
HC-1550-02	Hollow core	400	3	400	N.A.	25

**Table 2:** Temperature sensitivity of LPGs.

Fiber Model	Mode	S_T_ (pm/°C)	R^2^
SMF28	$\lambda_{3}$	50.6	0.9996
PS1250/1500	$\lambda_{3}$	−265.1	0.9987
P-SM-5	$\lambda_{3}$	−58.0	0.9827
DrakaSRH	$\lambda_{3}$	29.8	0.9985
PM1300-XP	$\lambda_{1}^{\left(F\right)}$	76.8	0.9990
	$\lambda_{2}^{\left(F\right)}$	77.7	0.9989
	$\lambda_{3}^{\left(F\right)}$	96.3	0.9999
	$\lambda_{1}^{\left(S\right)}$	~0	-
	$\lambda_{2}^{\left(S\right)}$	−25.6	0.9807
	$\lambda_{3}^{\left(S\right)}$	−34.9	0.9921
HC-1550-02	$\lambda_{1}$	11.9	0.9972
	$\lambda_{2}$	13.8	0.9994

**Table 3:** Strain sensitivity of LPGs.

Fiber Model	Mode	S_ε_ (pm/µε)	R^2^
PM1300-XP	$\lambda_{1}^{\left(F\right)}$	−1.57	0.9993
	$\lambda_{2}^{\left(F\right)}$	−1.51	0.9985
	$\lambda_{3}^{\left(F\right)}$	−1.82	0.9991
	$\lambda_{1}^{\left(S\right)}$	0.57	0.9990
	$\lambda_{2}^{\left(S\right)}$	1.41	0.9967
	$\lambda_{3}^{\left(S\right)}$	1.47	0.9992
HC-1550-02	$\lambda_{1}$	−0.82	0.9988
	$\lambda_{2}$	−0.72	0.9991
